# Biomimetic Surface Engineering of Ti-15Zr (Roxolid™) Implants: Enhancing Osseointegration and Bone Regeneration—A Comprehensive Review

**DOI:** 10.3390/biomimetics11070471

**Published:** 2026-07-06

**Authors:** Antonio Libonati, Danilo Marroni, Giulio Barbalace, Giulia Campanella, Carla Clemente, Francesco Campanella, Lucrezia Secreti, Vincenzo Campanella

**Affiliations:** 1 Dental School, Catholic University of Our Lady of Good Counsel, 1000 Tirana, Albania; 2 Dental School, University of Rome “Tor Vergata”, 00133 Rome, Italy; 3 Department of Clinical Sciences and Traslational Medicine, Dental School, University of Rome “Tor Vergata”, 00133 Rome, Italy

**Keywords:** Ti-15Zr, Roxolid™, biomimetic surface engineering, osseointegration, osteoinstruction, bone regeneration

## Abstract

Titanium-based dental implants have evolved significantly, with the development of binary alloys like Ti-15Zr (Roxolid™) representing a pivotal advancement in mechanical performance. Current research focuses on biomimetic surface engineering to further accelerate osseointegration and optimize bone regeneration, particularly in clinically compromised sites. This review constitutes a narrative synthesis of how these strategies replicate the bone extracellular matrix (ECM) through a holistic framework of architectural, mechanical, and biochemical integration. A structured literature search across PubMed, Scopus, and Web of Science (2010–2026) identified relevant studies focusing on the synergy between Ti-15Zr substrates and surface modifications. Evidence confirms that the high fatigue strength of Roxolid™ alloys provides an ideal foundation for advanced, hierarchical surface engineering without compromising structural integrity. This strategy utilizes macro-topography for primary stability, nano-topography for protein adsorption, and bio-functionalization (e.g., RGD peptides and osteogenic ions) to direct mesenchymal stem cell (MSC) differentiation. This synergy accelerates the transition from passive to active osseointegration, effectively bridging the “biological gap” during early healing. Biomimetic engineering transforms implants into instructive biological platforms, improving outcomes for patients with compromised bone quality and facilitating predictable immediate loading protocols.

## 1. Introduction

The selection of biomaterials for dental implants has evolved significantly to address the requirements of both osseointegration and long-term mechanical stability. Titanium (Ti) and its alloys remain the standard in implant dentistry due to their favorable biocompatibility and mechanical robustness [1,2]. Among these, the binary titanium–zirconium (Ti-Zr) alloy, specifically Roxolid™, has gained clinical relevance because it offers higher fatigue strength compared to commercially pure titanium (cpTi) without compromising biological integration [3,4].

While the mechanical benefits of Ti-Zr are well-documented, the clinical success of dental implants depends on the rapid establishment of a stable interface with the surrounding bone. The biological sequence—starting with blood protein adsorption, followed by inflammatory cell infiltration and subsequent osteogenic differentiation—is critical for implant success [5,6]. Despite the inherent biocompatibility of Ti-Zr, there is ongoing interest in optimizing surface characteristics to accelerate healing cascade, particularly in compromised clinical scenarios such as diabetic patients or those with low bone density [7,8].

Current research has shifted toward biomimetic surface engineering to modulate the peri-implant microenvironment. Various techniques, including the chemical immobilization of adhesive peptides (e.g., RGD sequences) and the incorporation of bioactive glasses or inorganic ions, have been investigated to influence cell signaling and immune responses [9,10]. These modifications aim to bridge the gap between static titanium surfaces and dynamic bone tissue, potentially enhancing the transition from the inflammatory phase to the proliferative phase of healing.

This paper provides a comprehensive overview of the current understanding of Ti-Zr implants. It synthesizes existing evidence on the mechanical properties of Ti-Zr alloys and critically evaluates recent advancements in biomimetic surface modifications, with a specific focus on how these interfaces regulate the molecular pathways involved in osseointegration. For the purpose of this review, we established clear definitions for the following key terms to ensure consistency: “biofunctionalization” refers specifically to the chemical/physical surface modification process; “osteoinstructivity” describes the biological cell–material interaction; “osseointegration kinetics” refers to the temporal measurements of bone formation; and “biomimetic engineering” is used as the overarching framework for these strategies. To ensure clarity and consistency throughout this review, we established specific definitions for key technical terms. We define “biofunctionalization” as the chemical or physical modification process; “osteoinstructivity” describes the biological cell–material interaction; “osseointegration kinetics” refers to temporal measurements of bone formation; and “biomimetic engineering” is used as the overarching framework for these strategies.

## 2. Materials and Methods

A structured literature search was conducted to provide a comprehensive overview of the current status of biomimetic surface engineering on Roxolid™ implants. This format provides a critical and integrative synthesis of the available evidence, combining data from preclinical, translational, and clinical studies. The methodology focused on identifying relevant studies published between January 2010 and March 2026, ensuring the evolution of the field was covered from early mechanical modifications to current bio-instructive surface technologies. The search was performed across PubMed, Scopus, and Web of Science, using the following search string:

(“Ti-15Zr” OR “Roxolid”) AND (“Biocompatibility” OR “Cell Viability”) AND (“Surface treatment” OR “SLA ACTIVE” OR “SLA” OR “Biomimetic Surface” OR “Surface Engineering”)

Additional hand-searching was performed on reference lists of included articles and leading implantology journals to ensure comprehensiveness. Boolean operators were used to refine the search and combine the selected concepts appropriately. No language restrictions were applied during the initial search.

### 2.1. Eligibility Criteria

**Inclusion Criteria are listed as follows:** (1) Peer-reviewed articles focusing on Ti-Zr (Roxolid™) implants; (2) studies investigating surface modifications (physical, chemical, or biological, e.g., anodization, functionalization, peptide coating); (3) articles discussing the impact of surface engineering on cellular response (e.g., osteoblast differentiation and protein adsorption) or bone regeneration; (4) studies comparing Ti-Zr alloys to CP Grade 4 Titanium; (5) both in vitro (cell-based) and in vivo (animal/clinical) studies.

**Exclusion Criteria are listed as follows:** (1) studies focusing solely on standard titanium (Grade 4) without comparative relevance to Ti-Zr alloys; (2) grey literature (e.g., conference abstracts without full-text documentation); (3) research with insufficient details on surface characterization methodologies.

### 2.2. Study Selection and Data Extraction

The search yielded an initial pool of 954 articles. Following screening of titles, abstracts, and full texts against the eligibility criteria, 53 studies met the inclusion criteria of this review. Selected full-text articles were analyzed to extract qualitative data regarding surface modification techniques (e.g., SLA and anodization) and characterization (SEM, XPS and contact angle); mechanical-biological synergy (cell viability, differentiation markers—RUNX2, ALP—and in vivo bone-to-implant contact percentages); and clinical outcomes. This comprehensive approach allowed for a critical narrative synthesis of the literature, highlighting both established evidence and future challenges in the field of biomimetic surface engineering.

### 2.3. Methodological Quality Assessment

The methodological rigor of the included studies was evaluated qualitatively based on study design, reproducibility of surface characterization, suitability of the outcome measures, and consistency of the reported findings. Particular attention was paid to the translational relevance of the evidence, a clear distinction was made between mechanistic in vitro investigations, animal studies, and clinical observations. This approach enabled the integration of heterogeneous evidence while preserving scientific rigor and interpretative coherence.

### 2.4. Quality Assessment

The quality of the included studies was systematically assessed to evaluate the risk of bias and robustness of the reported data. For clinical studies, we employed the Newcastle–Ottawa Scale (NOS) to evaluate the methodological quality of non-randomized studies. For in vitro and experimental studies, we used a custom checklist based on established criteria, including sample size determination, use of appropriate controls, blinding of analytical procedures, and statistical power analysis. Each study was reviewed independently by two authors, and any discrepancies in quality scoring were resolved through discussion to ensure consensus.

## 3. Relevant Sections

The field of oral implantology is undergoing a fundamental transition from passive, bioinert structural replacement toward biomimetic, osteoinstructive interfaces that replicate the hierarchical architecture and physicochemical properties of the native dentoalveolar complex. This shift is motivated by the inherent limitations of conventional osteoconductive surfaces, which serve as inert scaffolds permitting cell migration and adhesion without actively modulating cellular behavior [11]. Osseointegration in this model depends entirely on the proximity and viability of host osteogenic cells, establishing merely a permissive—rather than instructive—biological milieu [12].

In contrast, osteoinstructive biomimetic implants are engineered to assume a proactive biological function [13,14]. By emulating the architecture and biochemical signature of the native bone extracellular matrix (ECM), these interfaces provide precise molecular and structural cues—via nanotopography and localized ions or bioactive molecule release—that actively drive the recruitment, proliferation, and differentiation of mesenchymal stem cells (MSCs) into mature, functional osteoblasts [15]. This molecular dialogue enables the host to recognize the implant as endogenous tissue rather than a foreign substrate, transforming the interface from a static contact zone into a dynamic site of accelerated bone regeneration [16]. The clinical relevance of this transition is particularly pronounced in high-risk cohorts—such as geriatric patients and individuals with systemic comorbidities such as diabetes or osteoporosis—in whom compromised bone metabolism substantially reduces the predictability of conventional implant therapy [17,18].

### 3.1. The Roxolid™ Implant System

Introduced by Straumann in 2009, Roxolid™ represents a significant paradigm evolution in implantology. This material was engineered to overcome the mechanical limitations of commercially pure titanium (cpTi) while maintaining superior biocompatibility. Roxolid™ is a binary, single-phase α-alloy composed of approximately 85% titanium (Ti) and 15% zirconium (Zr). Zirconium acts as a solid-solution strengthener, significantly increasing the mechanical properties of the alloy without introducing cytotoxic elements such as aluminum or vanadium, which have been associated with local irritation and potential systemic concerns in Ti-6Al-4V alloys [19,20,21].

The unique microstructural configuration of Ti-15Zr is central to its performance. Traditional Grade 4 cpTi exhibits grain sizes of 20–30 µm, with a larger and more equiaxed (polygonal) morphology characteristic of fully recrystallized titanium. In Ti-15Zr (Roxolid™), grain refinement is dramatic: grains are ultra-fine (approximately 1–2 µm), elongated, and densely packed, forming microbands as a direct result of the intensive cold-working process used. This process creates a very high dislocation density and a pronounced crystallographic texture, with basal planes oriented parallel to the wire axis—a configuration that confers increased mechanical strength without sacrificing toughness or ductility [22].

### 3.2. Mechanical Performance

This microstructural refinement translates into significant mechanical advantages. Roxolid™ exhibits a tensile strength that is 10–15% higher than Grade 4 cpTi, with yield strength values of approximately 799 MPa (SLA surface) and 784 MPa (machined surface), and ultimate tensile strength values of approximately 968 MPa and 987 MPa, respectively [23]. The alloy demonstrates a fatigue endurance limit approximately 30% higher than Grade 4 cpTi (560 MPa for machined surfaces and 500 MPa for SLA surfaces), ISO 14801 testing confirmed improvements in fatigue strength of 11–38% over Grade 4 counterparts depending on implant design. Fracture toughness remains slightly superior (~22.34 J/cm^2^ vs. ~19.30 J/cm^2^), with uniform elongation ductility maintained at ~6.0–6.2%.

**Figure 1:**
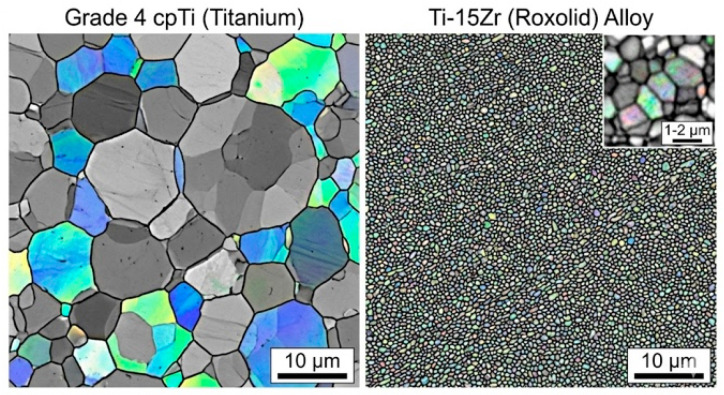
Visualization of the orientations of individual crystal grains within the metal structure of Roxolid™ (**right**) compared to cpTitanium Grade 4 (**left**).

Critically, Roxolid™ has a lower elastic modulus than cpTi, which helps mitigate the stress shielding effect—a mismatch between implant and bone stiffness that leads to peri-implant bone resorption [24]. The addition of zirconium improves the stability of the protective oxide layer, rendering Ti-Zr alloys more resistant to pitting corrosion in biological fluids [25,26]. This “mechanical surplus” is clinically decisive: it enables the development of implants with a narrower diameter (Ø 2.9–3.5 mm) that maintain structural integrity under high masticatory loads, frequently obviating the need for invasive bone augmentation procedures in sites with limited bone volume [27,28], Figure 1.

To contextualize the mechanical advantages of the Ti-15Zr alloy against the current scientific landscape, Table 1 provides a comparative overview of its key mechanical properties alongside standard clinical alternatives and emerging experimental biomaterials. As illustrated, Ti-15Zr offers a superior balance: it matches or exceeds the ultimate tensile and fatigue strengths of standard alloys while maintaining a lower elastic modulus, thereby mitigating stress-shielding risks.

### 3.3. Osseointegration: Biological Cascade and Molecular Mechanisms

Osseointegration constitutes the fundamental biological prerequisite for the long-term clinical success of endosseous implants. It is defined as the direct structural and functional connection between living, organized bone and the surface of a load-bearing implant, which is established and maintained without the interposition of fibrous connective tissue. This process is not a single event, but a temporally and spatially coordinated biological sequence that unfolds across overlapping phases, each governed by a distinct cellular and molecular program.

The cascade is initiated immediately upon surgical implant placement, when disruption of the cortical and medullary vasculature triggers the coagulation cascade and the formation of a provisional fibrin clot at the implant interface. This clot serves as a three-dimensional provisional scaffold enriched with growth factors—including platelet-derived growth factor (PDGF) and transforming growth factor-beta (TGF-β)—that orchestrate the subsequent recruitment of immune and progenitor cells. Concurrently, plasma proteins adsorb rapidly onto the implant oxide surface within seconds of blood contact, establishing a proteinaceous conditioning film that determines the nature of subsequent cell-surface interactions [29].

The acute inflammatory phase (0–3 days) is characterized by the sequential infiltration of neutrophils and monocyte-derived macrophages. Rather than a merely defensive response, this phase has an indispensable instructive role: macrophages adopting the pro-inflammatory M1 phenotype secrete cytokines—including IL-6, IL-1β, and TNF-α—that amplify the local inflammatory signal and prime the regenerative microenvironment [30]. Critically, the surface physicochemical properties of the implant directly modulate the macrophage activation state, establishing an immunological set-point that influences all subsequent healing events [31,32].

As the inflammatory phase resolves, the proliferative phase (~days 4–14) is marked by the transition of macrophages toward the anti-inflammatory, reparative M2 phenotype—a shift essential for pro-osteogenic milieu [33]. MSCs, recruited from the periosteum, endosteum, and circulating blood, undergo proliferation and osteoblastic commitment under the influence of Bone Morphogenetic Proteins (BMPs) and Wnt/β-catenin signaling. Concurrently, VEGF-driven angiogenesis restores the local oxygen and nutrient supply, a prerequisite for the energetically demanding process of mineral matrix deposition. Activated osteoblasts begin secreting an unmineralized collagenous osteoid directly onto the implant surface, initiating contact osteogenesis [34,35].

**Figure 2:**
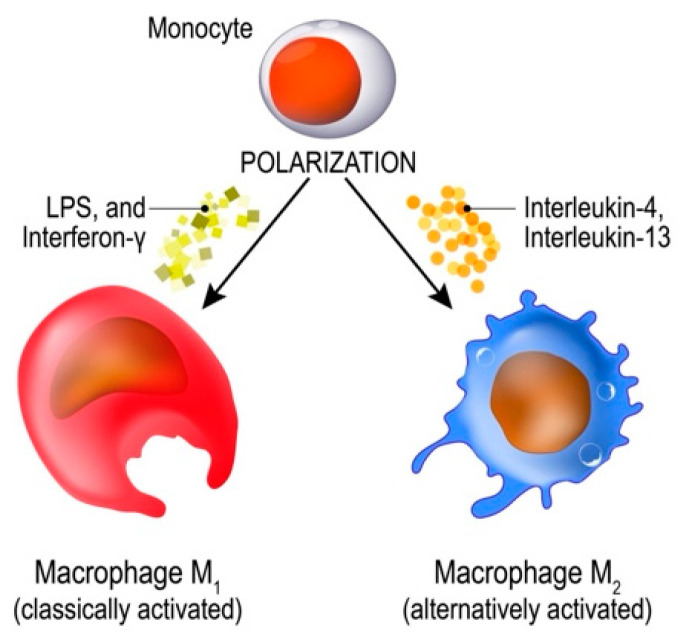
This schematic represents the Activation and Polarization of the Macrophage. (Source: Authors’ original illustration).

**Figure 3:**
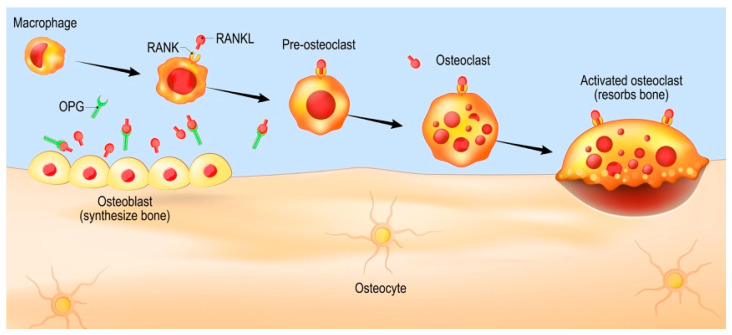
Bone Biology and Remodeling: the Role of RANK, RANKL and OPG. (Source: Authors’ original illustration).

The maturation and mineralization phase (days 14–21) is characterized by the progressive calcification of the osteoid matrix, evidenced biochemically by upregulation of Alkaline Phosphatase (ALP) activity and Osteocalcin (OC) expression [36,37], Figure 2. The final remodeling phase, extending over months to years, is governed by the RANKL/RANK/OPG signaling axis, which maintains the homeostatic equilibrium between osteoclast-mediated resorption and osteoblast-mediated apposition. Through iterative cycles of bone turnover, the initial woven bone is progressively replaced by organized lamellar bone with a mature Haversian architecture, increasing the BIC ratio and ensuring long-term biomechanical competence [38], Figure 3.

### 3.4. Surface Treatments and Biomimetic Functionalization

**Figure 4:**
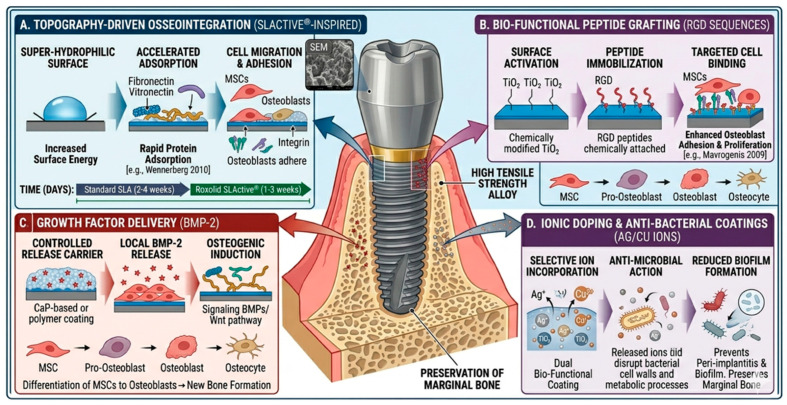
Biomimetic Advancements in Roxolid^TM^ Implant Surface Modifications (Source: Authors’ original illustration).

The surface of a dental implant comprises the primary determinant of the biological events governing osseointegration. Surface modification strategies are therefore fundamental engineering parameters that define the immunological, proteomic, and osteogenic trajectory of the healing response. These strategies are conventionally classified into three mechanistically distinct categories—subtractive (physical), additive (chemical and coating-based), and biomolecular (biofunctional)—each operating at a distinct stage of the biological cascade [39,40], Figure 4.

While categorizing these modifications is conceptually useful, their critical translation to clinical practice requires an evaluation of the specific manufacturing procedures, their functional advantages, and their inherent technical limitations. For instance, while subtractive methods like SLA are highly predictable, they lack active biological signaling; conversely, advanced additive and biofunctional methods offer high osteoinstructivity but face challenges of mechanical delamination under shear stress, complex manufacturing scalability, and regulatory hurdles. To provide a comprehensive and critical evaluation, Table 2 summarizes the specific fabrication procedures, primary advantages, and notable limitations of the key surface treatments applied to Ti-Zr implants.

#### 3.4.1. Subtractive Modifications: SLA and SLActive^®^

Subtractive surface modifications operate by selectively removing material from the implant surface to generate controlled topographic features. Sandblasting with large-grit alumina or silicon carbide particles (typically 250–500 µm) creates macro-scale surface irregularities (20–40 µm) that enhance mechanical interlocking with the host bone. Subsequent acid etching—most commonly with hot HCl/H_2_SO_4_ mixtures—superimposes a finer micro-scale texture (2–5 µm pits) that promotes fibrin network entrapment, platelet activation, and osteoblast adhesion. The resulting bimodal **SLA^®^** topography represents the current clinical gold standard [41].

In the context of Roxolid™ implants, the **SLActive^®^** (modSLA) surface represents a further evolution. These are produced by storing the acid-etched implant under a nitrogen atmosphere in isotonic saline to prevent hydrocarbon contamination. This results in superhydrophilicity (contact angle ≈ 0°) and spontaneous formation of nanostructures not present on conventional SLA surfaces, as revealed by high-magnification SEM imaging. These nanostructures catalyze fibrin network formation within 15 min of blood contact, reducing the standard healing period from 6–12 weeks to approximately 3–4 weeks and enabling earlier prosthetic loading [42]. Furthermore, SLA treatment of Ti-Zr substrates modulates the proteomic adsorption profile of proteins from saliva and plasma—enriching functionally relevant species such as serum albumin and fibronectin—thereby influencing the dynamics of initial cell adhesion and bacterial biofilm formation [43].

#### 3.4.2. Additive Modifications: Anodization and Calcium Phosphate Coatings

Additive surface modifications introduce new chemical phases or topographic features onto the implant surface. Anodic oxidation (anodization) is the most widely employed electrochemical technique, producing a thickened, crystalline TiO_2_ layer enriched with a self-organized nanotubular architecture. These nanotubes—with diameters tunable between 20 and 200 nm depending on electrolyte composition and applied voltage—dramatically increase the surface-to-volume ratio, enhance protein adsorption kinetics, and serve as reservoirs for the controlled local release of pharmacological agents [44]. Hydroxyapatite (HA) and calcium phosphate (CaP) coatings, deposited by plasma spraying, biomimetic precipitation, or pulsed laser deposition, introduce a mineral phase chemically analogous to the inorganic component of bone, establishing a direct osteoconductive interface. The susceptibility of plasma-sprayed HA coatings to delamination under cyclic mechanical loading has driven the development of thin-film nanocrystalline HA deposition techniques that preserve adhesive strength while maintaining osteogenic bioactivity [35,43].

#### 3.4.3. Biofunctional Modifications: The Five Biomimetic Axes

The most advanced category encompasses biofunctional surface modifications, which transition the implant from a passive scaffold into an active biological signaling platform. The biological rationale derives from native bone ECM, which integrates a hierarchical nano- to microscale architecture—primarily composed of type I collagen fibrils and carbonated hydroxyapatite crystallites—with a biochemical repertoire of adhesion ligands, sequestered growth factors, and mechanotransductive signals that collectively govern cell adhesion, migration, proliferation, and differentiation. Contemporary biomimetic strategies are organized along five principal axes:

**Axis 1: ECM-mimetic coatings.** Structural proteins such as collagen and glycosaminoglycans like hyaluronic acid are deposited on the implant surface to recreate the adhesive and viscoelastic properties of the pericellular matrix, promoting osteoprogenitor cell attachment and directional migration.

**Axis 2: Molecular and ionic functionalization.** At the peptide level, the covalent or adsorptive immobilization of RGD (Arg-Gly-Asp) and related sequences such as GRGD directly engages α_5_β_1_ and αvβ_3_ integrins on MSC membranes, activating FAK/ERK and PI3K/Akt signaling cascades that converge on osteogenic transcription factors, most notably RUNX2. The P-15 peptide—a synthetic analog of the cell-binding domain of type I collagen—further accelerates early osseointegration [45]. At the ionic level, incorporation of Sr^2+^ and Mg^2+^ within the surface oxide layer modulates bone remodeling: Sr^2+^ simultaneously promotes osteoblast-mediated formation and downregulates osteoclastic resorption through modulation of the RANKL/OPG ratio and activation of the Wnt/β-catenin pathway, while Mg^2+^ acts as a cofactor for integrin-mediated cell adhesion and stimulates peri-implant angiogenesis via VEGF upregulation [46].

**Axis 3: Growth factor tethering.** Controlled-release incorporation of Bone Morphogenetic Proteins (BMP-2, BMP-4, BMP-7) activates SMAD-dependent pathways to drive MSC commitment toward the osteoblastic lineage; PDGF and VEGF coordinate the angiogenic response essential for sustaining the metabolic demands of active bone formation [47].

**Axis 4: Bioactive ceramic functionalization.** Nanocrystalline hydroxyapatite establishes direct chemical continuity with the mineral phase of host bone, promoting rapid interfacial bonding and reducing the critical lag period between implant placement and functional loading [48,49].

**Axis 5: Antibacterial functionalization.** Since bacterial biofilm formation at the transmucosal interface is the primary etiological driver of peri-implantitis, surface strategies incorporating drug-coated platforms (antibiotics, bisphosphonates, and statins), antimicrobial peptides such as GL13K, and stimuli-responsive systems—including zinc or silver ion release and pH-responsive drug delivery—represent an integrated approach that simultaneously addresses osseointegration and infection prevention [50].

The ideal biomimetic surface modification should be resorbable at a physiologically controlled rate, immunologically inert, mechanically stable under functional loading, and capable of providing spatiotemporally appropriate biological cues without inducing ectopic or dysregulated tissue responses.

### 3.5. Hierarchical Multi-Scale Engineering of the Tissue-Implant Interface

Modern endosseous implantology is characterized by the hierarchical, multi-scale engineering of the tissue-implant interface. Surface design is conceptualized as a stratified framework in which each level of modification optimizes the biological response at a distinct stage of the healing cascade—from initial mechanical anchorage to long-term molecular osseointegration [51], Table 3.

#### 3.5.1. Macro- and Micro-Topography

Macro-topography—defined by thread geometry and overall implant morphology—ensures primary mechanical interlocking with the surrounding bone, constituting a prerequisite for immediate and early loading protocols [52]. At the macroscale (10 µm to mm), implant geometry governs primary mechanical stability through physical interlocking with the surrounding bone; at the microscale (1–10 µm), surface features maximize BIC and promote mechanical interdigitation with mineralized tissue [53]. The SLA process creates a bimodal topography: large-grit sandblasting (corundum/Al_2_O_3_) generates macro-roughness (20–40 µm), while subsequent hot acid etching (HCl/H_2_SO_4_) superimposes micro-pits (2–5 µm) that trap fibrin networks and promote osteoblast adhesion.

Surface roughness, quantified by Sa (arithmetic mean height) and Sz (maximum peak-to-valley height), is a principal modulator of the host cellular response [54]. Moderately rough surfaces (Sa 1–2 µm) are currently considered optimal for the osteogenic response. Although increased roughness has been theoretically associated with higher susceptibility to bacterial colonization, prospective clinical trials have not demonstrated a significantly elevated incidence of peri-implantitis for implants within the moderately rough range [55]. Surface wettability—expressed as the water contact angle—governs protein adsorption kinetics and cell–surface interactions; hydrophilic surfaces promote the rapid adsorption of fibronectin and vitronectin, accelerating early tissue healing and osteoblast differentiation [56].

#### 3.5.2. Nano-Topography: Protein Adsorption and MSC Recruitment

Nano-topographic features (1–100 nm) serve as the primary interface for early biological signaling. By mimicking the nanostructure of the native ECM, these modifications enhance the adsorption of key serum proteins—principally fibronectin and vitronectin—which act as adhesion anchors for MSCs, triggering intracellular signaling cascades that direct osteoblastic differentiation. The most clinically relevant technique for generating nanostructures on Ti-Zr alloys is anodization, wherein controlled electrolytic oxidation with strong acids (H_2_SO_4_, H_3_PO_4_, or HF) produces a dense, self-organized array of TiO_2_ nanotubes. Advanced chemical etching with H_2_SO_4_/H_2_O_2_ has been used to create hierarchical Micro-/Submicro-/Nanostructured (MSN) surfaces on Ti-Zr alloys, demonstrating significantly higher hydrophilicity and osteoblast attachment compared to standard machined or mono-scale etched surfaces [57].

#### 3.5.3. Molecular Functionalization: Active Osteoinduction

Surface functionalization of Ti-15Zr implants with bioactive molecules, such as RGD (Arg-Gly-Asp) peptides, aims to mimic the bone extracellular matrix, thereby promoting rapid cell adhesion and proliferation. Studies have shown that utilizing innovative dip-coating techniques to immobilize RGD sequences on the titanium–zirconium oxide layer significantly enhances the biological response, facilitating early osteoblast recruitment and improved fibrin network formation at the implant interface [58,59]. **Ion incorporation:** Sr^2+^ and Mg^2+^ within the TiO_2_ nanostructure modulate local bone remodeling. In the Ti-Zr context, SLA treatment selectively enriches serum albumin and fibronectin from saliva and plasma, simultaneously enhancing osteoprogenitor cell recognition and attenuating bacterial biofilm formation. **Growth factors and drug delivery.** Surfaces can additionally be functionalized with antibiotics (e.g., gentamicin), analgesics, Ca-phosphate, or bisphosphonates for localized delivery, enhancing infection control and accelerating bone cell proliferation without systemic cytotoxic effects. While ion incorporation serves as a powerful strategy for modulating the biological response and enhancing antimicrobial activity, the release kinetics are of paramount importance. An uncontrolled ‘burst release’ of ions could potentially exceed the cytotoxicity threshold for osteoblasts and fibroblasts, leading to adverse local tissue reactions. Consequently, the challenge in biomimetic surface engineering lies in designing surfaces that provide a controlled, sustained ion release, effectively balancing the therapeutic antimicrobial benefit with long-term biocompatibility. Maintaining ion concentrations within this ‘therapeutic window’—high enough to inhibit bacterial colonization but low enough to avoid systemic toxicity—is a prerequisite for the clinical safety of Ti–Zr implants. Further longitudinal studies are warranted to assess the cumulative ion dosage in the peri-implant environment to ensure the safety and structural integrity of these modifications over the long term.

#### 3.5.4. Biomechanical Optimization: Stress Shielding and Structural Compliance

A critical limitation of conventional titanium implants is the mechanical mismatch with host bone. The higher stiffness of metallic substrates relative to cortical bone alters local strain distributions—a phenomenon termed stress shielding—leading to peri-implant bone resorption. The biomimetic approach addresses this through: (1) optimization of the implant’s Young’s modulus using low-modulus alloys (such as Ti-15Zr) or PEEK-based composites; and (2) the implementation of 3D-printed, porous lattice structures that permit deep tissue infiltration and physiological load transfer. By mirroring the hierarchical porosity of native trabecular bone, these architectures ensure long-term structural stability while preserving the integrity of the surrounding host environment [60,61].

The frontier of surface engineering further involves stimuli-responsive “smart” materials capable of dynamic modulation of surface chemistry in real time. pH-responsive antibacterial release and toggling between antimicrobial and osteogenic surface states represent the next generation of implant design [62,63].

### 3.6. The Ti-15Zr (Roxolid™) Alloy: Mechanical and Biological Rationale

The binary Ti-15Zr alloy (Roxolid™) exemplifies the integration of biomechanical superiority with advanced surface engineering capability [64,65]. By retaining the α-phase crystalline structure of cpTi while incorporating 15% zirconium, the alloy achieves a ~40% increase in tensile strength and fatigue resistance without compromising biocompatibility. This mechanical robustness enables the use of narrow-diameter implants (NDIs, Ø 3.3 mm) in anatomically challenging sites, frequently obviating the need for invasive bone grafting [66,67].

In pure titanium, aggressive treatments such as deep acid etching or heavy grit-blasting may introduce micro-cracks or compromise surface integrity; the improved fatigue resistance of Ti-15Zr permits more sophisticated surface engineering without such risks [68]. Furthermore, the native zirconium oxide (ZrO_2_) layer that forms on the alloy surface provides enhanced chemical stability and a superior base for biomimetic functionalization—including hydroxyapatite deposition, TiO_2_ nanotube growth, and bioactive peptide immobilization [69,70].

Roxolid™ is compatible with both SLA^®^ and SLActive^®^ surface technologies. Histomorphometric data from animal models confirm that modSLA Roxolid™ implants achieve higher BIC ratios and improved marginal bone level (MBL) stability, particularly in defect-grafted sites [71,72].

### 3.7. Biological Response: Immunomodulation, Osteoblast Activity, and Proteomic Profile

#### 3.7.1. Macrophage Polarization and Immunomodulation

The biological response to Roxolid™ surfaces is deeply governed by immunomodulatory mechanisms. Research demonstrates that Ti-Zr modSLA surfaces drive macrophage polarization toward the M2 phenotype more effectively than traditional titanium surfaces, suppressing chronic pro-inflammatory cytokine release (IL-6, TNF-α, IL-1β) and upregulating anti-inflammatory mediators (IL-10, TGF-β1), establishing a conducive microenvironment that allows osseointegration to commence earlier [73]. A related immunomodulatory strategy involves the coating of Roxolid™ surfaces with S53P4 bioactive glass, whose dissolution products significantly reduce pro-inflammatory cytokine expression in human gingival fibroblasts and osteoblasts, preventing excessive bone resorption and promoting a stable biological seal.

#### 3.7.2. Osteoblast Adhesion, Differentiation, and Proteomic Profile

In vitro studies confirm that osteoblast-like cells (MC3T3-E1) attach in significantly higher numbers to Ti-Zr surfaces than to cpTi, demonstrating increased ALP activity and elevated OC expression, indicative of mature mineralized matrix production [74]. These effects are potentiated by zirconium ions, which exert direct osteoinductive activity on osteoblast maturation. At the molecular level, SLA treatment on Ti-Zr results in a unique proteomic adsorption pattern from saliva, including 14 exclusive proteins—among them serum albumin—which mediate initial cell adhesion and may concomitantly reduce bacterial biofilm formation. Collagen- and hyaluronic acid-based ECM coatings further enhance soft-tissue sealing and MSC recruitment, while RGD peptide grafting onto the Roxolid™ surface significantly improves BIC and removal torque values.

### 3.8. Clinical Performance of Ti-15Zr Narrow-Diameter Implants

Systematic reviews and meta-analyses consistently confirm the high clinical predictability of Roxolid™ 3.3 mm NDIs, establishing them as a reliable alternative to bone grafting in atrophic bone regions. Reported survival rates exceed 98.4% at 1 year and 97.7% at 2 years, with MBL averaging 0.41 mm after 2 years—values comparable to, or exceeding, those of standard-diameter titanium implants [75]. No implant body fractures were reported across primary studies, validating the fatigue strength advantages of the Ti-Zr alloy. Long-term follow-up data further confirm that Ti-Zr SLA surfaces demonstrate significantly greater soft-tissue attachment compared to machined or ceramic surfaces, ensuring a robust biological seal against oral pathogens and reduced bleeding on probing (BoP) [76], Table 4 and Table 5.

### 3.9. Biomimetic Surface Engineering of Roxolid™ in Bone Regeneration: From Guided Protocols to Instructive Scaffolds

The biomimetic potential of Ti-15Zr (Roxolid™) extends beyond the immediate implant–bone interface to encompass the broader biological domain of bone regeneration, rendering it a uniquely versatile osteoinstructive platform in anatomically and systemically compromised clinical scenarios. This regenerative capacity is grounded in two converging material properties: a lower elastic modulus (approximately 96–99 GPa) relative to commercially pure titanium, which mitigates stress shielding by transferring mechanical loads more uniformly to the surrounding bone and thereby prevents disuse atrophy while sustaining healthy remodeling; and a hierarchically engineered surface that actively modulates the sequential biological cascade—hemostasis, inflammation, proliferation, and remodeling—governing peri-implant bone formation.

A cornerstone of this osteoinstructive capacity is immunomodulation. The physicochemical properties of the Ti-Zr surface, particularly when combined with superhydrophilic SLActive^®^ treatment, suppress chronic inflammation and establish a pro-osteogenic microenvironment essential for MSC recruitment and osteoblast commitment. Concurrently, the interaction of the Roxolid™ surface with biological fluids generates a distinctive proteomic adsorption profile: the presence of zirconium selectively enriches proteins implicated in cell adhesion and coagulation cascade initiation—the primary biological triggers of the healing cascade—thereby priming the interface for accelerated tissue integration.

Biomolecular functionalization further transforms the Ti-15Zr surface into an active regenerative platform. At the ionic level, Sr^2+^ doping simultaneously promotes osteoblast-mediated bone formation and suppresses osteoclastic resorption through modulation of the RANKL/OPG ratio, while Mg^2+^ acts as a cofactor for integrin-mediated adhesion and stimulates peri-implant angiogenesis via VEGF upregulation. Notably, copper (Cu^2+^)-doped Ti-Zr alloys confer intrinsic antibacterial properties—demonstrating up to 89% inhibition of adherent *Porphyromonas gingivalis*—without compromising cytocompatibility, representing a compelling multifunctional strategy that unifies osseointegration, angiogenesis, and infection control within a single surface architecture.

These surface-level properties translate directly into measurable regenerative advantages in both guided bone regeneration (GBR) protocols and scaffold-based strategies. In GBR applications, the mechanical surplus of Roxolid™ narrow-diameter implants enables simultaneous placement with resorbable or non-resorbable membranes and bone substitutes in sites of severe ridge atrophy, sustaining functional loads in grafted, low-density bone without compromising structural integrity [77]. The hierarchical surface engineering synergizes with the regenerative milieu established by the membrane barrier, accelerating MSC recruitment into the newly forming bone compartment; preclinical minipig models confirm that superhydrophilic Roxolid™ implants achieve significantly higher bone-to-implant contact (BIC) and faster bone apposition compared to standard SLA titanium controls, even in circumferential defects grafted with bone substitutes. At the scaffold level, three-dimensional-printed, patient-specific Ti-Zr porous lattice structures—designed to replicate the hierarchical porosity of native trabecular bone (pore sizes 300–600 µm, interconnectivity >80%)—enable deep vascular ingrowth, physiologically compliant load transfer, and direct surface functionalization with hydroxyapatite or bioactive peptides on the scaffold struts. The native ZrO_2_-enriched oxide layer characteristic of Ti-15Zr further provides a chemically superior substrate for biomimetic calcium phosphate precipitation and BMP-2 tethering relative to cpTi, yielding significantly higher bone volume fraction (BV/TV) and earlier mineralization onset in critical-size defect models.

Clinical translation corroborates these preclinical findings. Survival rates for 3.3 mm Roxolid™ implants exceed 98.4% at one year and 97.7% at two years, with superhydrophilic surfaces reaching maximum secondary stability approximately twice as rapidly as conventional SLA surfaces—critically compressing the vulnerability window of the stability dip at weeks 2–4 of healing. Beyond bone, Ti-Zr SLActive^®^ surfaces demonstrate superior soft-tissue attachment relative to machined or ceramic counterparts, establishing a robust transmucosal biological seal that protects the underlying regenerated bone from bacterial infiltration and the sequelae of peri-implantitis [78]. Collectively, the integration of stress-shielding mitigation, immunomodulatory surface chemistry, multifunctional biomolecular functionalization, and scaffold-level architectural biomimicry positions Ti-15Zr as a uniquely comprehensive osteoinstructive platform—one whose regenerative potential extends well beyond conventional osseointegration to address the full biological complexity of bone regeneration in the most demanding clinical environments, including post-oncological defects and patients with systemic conditions compromising bone metabolism.

## 4. Discussion

The current findings underscore a fundamental transition in oral implantology, where the convergence of subtractive and additive surface engineering represents the frontier of biomimetic design. The integration of both modalities is essential for transitioning from bioinert, osteoconductive substrates to highly osteoinstructive interfaces that actively modulate the host response, particularly in patients with compromised metabolic or regenerative capacity. The clinical success of Roxolid™ implants in challenging scenarios is not solely attributable to mechanical strength, but to a sophisticated synergy between material science and biomimetic surface engineering.

While standard Grade 4 titanium implants often require a specific diameter threshold to ensure structural reliability, the superior fatigue strength of Ti-15Zr provides a “mechanical surplus” that facilitates advanced surface engineering without risking mechanical failure. Recent studies confirm that aggressive modifications—such as electrochemical anodization to create nanotubes or the application of nano-coatings—do not compromise the structural stability of the Ti-Zr body, unlike in pure Ti implants [79].

This hierarchical modification strategy operates across three distinct tiers: the macro/micro-level, where SLA remains the foundational anchor for mechanical interlocking; the nano-level, where nanofeatures mimic the natural architecture of the bone ECM to enhance serum protein adsorption and MSC recruitment; and the molecular-level, where functionalization with RGD peptides, osteogenic ions (Ca^2+^, Mg^2+^, Sr^2+^), and growth factors actively instructs osteoblasts. Mg^2+^ acts as a cofactor for integrin-mediated cell adhesion, while Sr^2+^ exerts a dual-action effect of increasing osteoblastic activity while simultaneously downregulating osteoclastic resorption through RANKL/OPG ratio modulation. This osteoinstructive potential effectively compensates for host regenerative deficiencies in compromised environments such as atrophic or D4-type bone, transforming previously unfavorable sites into responsive biological beds by stimulating Wnt/β-catenin and MAPK signaling pathways [80]. To contextualize the performance of the Ti-15Zr (Roxolid™) system within the broader landscape of contemporary implantology, it is essential to consider alternative high-performance biomaterials currently under investigation. While the α-phase Ti-15Zr alloy offers a compelling balance of enhanced fatigue strength and a reduced elastic modulus compared to Grade 4 cpTi, other research pathways have extensively focused on metastable β-titanium alloys, particularly Ti-Nb-based systems (such as Ti-Nb-Zr and Ti-Nb-Ta-Zr). These β-Ti formulations exhibit an even lower elastic modulus, often dropping to 55–70 GPa, which closely matches the mechanical compliance of human cortical bone and minimizes the risk of stress shielding [81,82]. However, their lower ultimate tensile strength compared to heavily cold-worked Ti-15Zr can restrict their predictability in narrow-diameter configurations that face extreme masticatory loads [83,84]. Concurrently, zirconia (ZrO_2_)-based ceramics—such as yttria-stabilized tetragonal zirconia polycrystals (Y-TZP) and alumina-toughened zirconia (ATZ)—have emerged as prominent metal-free alternatives. Zirconia implants provide outstanding aesthetic outcomes, excellent soft-tissue integration, and a low affinity for bacterial biofilm accumulation; nonetheless, their inherent brittleness and susceptibility to low-temperature degradation under cyclic fatigue remain critical challenges when contrasted with the high fracture toughness and structural reliability of the binary Ti-15Zr alloy [85,86]. Therefore, while these emerging low-modulus alloys and ceramic materials represent promising frontiers in biomaterials science, the Ti-15Zr system currently strikes a highly pragmatic clinical balance, providing the mechanical surplus required for minimally invasive protocols while serving as an ideal, stable substrate for advanced biomimetic surface modifications.

A primary debate in the literature concerns the risk of “over-engineering” the surface. However, it is demonstrated that the superior fatigue limits of Roxolid™ provide a significant safety margin for highly advanced bio-functionalization without compromising long-term stability. Functionalized Ti-Zr surfaces significantly shorten the “biological gap” between initial stabilization and functional integration, representing a paradigm shift from mechanical to biological loading protocols that may eventually allow for immediate functional loading regardless of bone quality, provided the surface is sufficiently “instructed.”

Despite the considerable advances described, several limitations warrant acknowledgment. Much of the evidence for osteo-instructive surfaces is derived from in vitro and animal models, and the stability of bioactive coatings under cyclic masticatory forces remains an area where robust clinical validation is still needed. The current body of clinical evidence exhibits significant heterogeneity in surface characterization protocols, cell culture conditions, and trial methodologies, complicating direct inter-study comparisons and rendering the establishment of a universally accepted “gold standard” surface specification elusive. Future research should prioritize the standardization of surface characterization parameters—particularly Sa, Sz, contact angle, and protein adsorption profiles—alongside prospective randomized controlled trials with extended follow-up periods and harmonized outcome measures, with particular attention to systemically compromised patient populations. The clinical translation of stimuli-responsive smart surfaces, dynamic drug delivery systems, and AI-assisted implant design represents the most promising frontier for the next generation of biomimetic implantology.

### 4.1. Mechanical Properties

Beyond traditional load-bearing capacity, a deeper understanding of mechanical performance requires consideration of surface hardness and the coefficient of thermal expansion (CTE). Hardness is a key indicator of the material’s resistance to localized plastic deformation and surface wear, which is particularly relevant during the insertion process and in the presence of surface modifications. Furthermore, for surface-engineered implants, the CTE compatibility between the Ti–Zr substrate and the deposited coating is critical. A significant mismatch in CTE values can induce localized thermal stress during cooling processes or intraoral temperature fluctuations, potentially leading to coating delamination and loss of structural integrity at the interface. Therefore, ensuring CTE matching is a prerequisite for maintaining the long-term performance and durability of biomimetic surface modifications.

### 4.2. Current Limitations and Future Perspectives

Despite the promising potential of biomimetic surface modifications, several challenges must be addressed before widespread clinical implementation. From a **regulatory perspective**, the standardization of characterization protocols remains a significant hurdle, as diverse testing methodologies complicate the comparison between different materials. **Translational and manufacturing challenges** represent a second critical bottleneck; while many surface modifications show excellent results in controlled laboratory settings, scaling these technologies for industrial-grade production—while maintaining consistency, sterility, and cost-effectiveness—is not trivial.

Furthermore, there is a clear **lack of long-term clinical data**. Most current evidence is derived from pre-clinical models or short-term follow-up studies. Future research should prioritize well-designed, multicenter, long-term clinical trials to evaluate the real-world performance of these surfaces. Addressing these limitations is essential to transition biomimetic implants from experimental success to standard clinical practice.

## 5. Conclusions

The integration of biomimetic surface engineering on Ti-15Zr (Roxolid™) substrates marks a pivotal evolution in modern implant dentistry. This transition from passive, bio-inert devices to active, osteo-instructive platforms redefines the biological dialogue at the implant–bone interface. The inherent mechanical superiority of Roxolid™ serves as an essential foundation, providing the structural surplus required to implement complex, hierarchical surface modifications—spanning from macro-mechanical anchoring to molecular bio-functionalization—without compromising long-term fatigue resistance or implant longevity. This hierarchical synergy effectively accelerates the healing cascade and improves clinical outcomes, particularly in patients with compromised bone quality.

The biomimetic approach holds significant promise for improving outcomes in patients with compromised bone quality and facilitating predictable immediate loading protocols. Clinical survival rates exceed 98.4% at one year and 97.7% at two years, with marginal bone loss averaging 0.41 mm, validating the biomechanical and biological rationale of the Ti-15Zr system. By unifying hierarchical topography, biomechanical compliance, and dynamic biochemical signaling, clinicians can generate a peri-implant environment recognized as “self” by the host tissue, promoting favorable immunomodulation and superior clinical success rates exceeding 98%. Ultimately, the integration of high-strength alloys with superhydrophilic nanostructured surfaces is poised to become the new gold standard, facilitating less invasive surgical interventions in atrophic bone and enabling more efficient, patient-centered immediate loading protocols across diverse and challenging clinical scenarios. Regarding the fabrication of Ti–Zr implants, the choice of manufacturing technique significantly dictates the final material properties. **Computer Numerical Control (CNC) machining** is currently the gold standard, providing implants with near-theoretical density and excellent fatigue strength, which are essential for long-term clinical success. Conversely, **Additive Manufacturing (AM)**, such as Selective Laser Melting (SLM), is increasingly explored to design implants with specific architectures.

The **optimal density** of the final product depends on the implant design: for bulk structural components, a density close to 100% is required to maximize mechanical performance and ensure the resistance to cyclic loading. However, in AM, controlled porosity (reduced density) can be intentionally introduced to lower the Young’s modulus—reducing stress shielding—and to promote secondary osseointegration through bone ingrowth. Thus, the selection of the manufacturing method must be balanced between the requirement for high density (mechanical integrity) and the strategic use of porosity (biological integration).

## 6. Future Directions

The field is rapidly evolving toward the development of “smart surfaces.” Emerging strategies, including the use of TiO_2_ nanotubes for controlled local drug-delivery systems, laser-structuring for optimized osteocyte interaction and antimicrobial resistance, and 3D-printed patient-specific Ti-Zr scaffolds, represent the next frontier in oral fixed rehabilitation. As molecular-level modifications and smart coatings continue to evolve, the predictability and longevity of Roxolid™ implants are expected to reach even higher standards. Future research should prioritize long-term clinical validation of bio-functional coatings under masticatory loading and the development of stimuli-responsive smart surfaces. Notwithstanding, future research must prioritize longitudinal clinical trials to validate the stability of bioactive coatings under sustained functional loads and to refine current osteo-instructive protocols, as the current literature remains heterogeneous and lacks definitive clinical evidence supporting the absolute superiority of any single specific surface design. While these results are promising, they should be interpreted with caution. Further long-term, high-quality clinical trials are warranted to definitively confirm these observations in a broader patient population.

## Figures and Tables

**Table 1:** Comparison of mechanical properties between Ti-15Zr (Roxolid™) and other contemporary implant biomaterials.

Biomaterial	Yield Strength (MPa)	Ultimate Tensile Strength (MPa)	Fatigue Endurance Limit (MPa)	Young’s Modulus (GPa)
Grade 4 cpTi (Current Standard)	~480–520	~550–680	~380–400	~105
Ti-6Al-4V (Grade 5 Alloy)	~ 860	~930	~550–600	~114
**Ti-15Zr (Roxolid™)**	~784–799	~968–987	500–560	96–99
β-Ti Alloys (e.g., Ti-Nb-Zr)	~400–600	~550–800	~300–400	55–70

**Table 2:** Overview of procedures, advantages, and limitations of main surface treatments on Ti-15Zr implants.

Surface Treatment	Fabrication Procedure	Primary Advantages	Main Limitations
**SLA (Subtractive)**	Blasting with large-grit alumina/corundum (250–500 µm) followed by hot acid etching (HCl/H_2_SO_4_).	Excellent primary stability; proven clinical track record; provides optimal macro/micro-roughness for mechanical interlocking.	Bio-inert (lacks active signaling); potential for residual blasting particles; slower initial protein adsorption compared to hydrophilic surfaces.
**SLActive/modSLA (Subtractive)**	SLA procedure followed by immediate storage in an isotonic saline solution under a nitrogen atmosphere to prevent hydrocarbon contamination.	Superhydrophilicity (contact angle ≈0°); accelerates fibrin network formation and initial osseointegration (3–4 weeks); enhances early MSC adhesion.	Higher manufacturing and packaging costs; highly sensitive to air exposure (loss of hydrophilicity if the liquid seal is broken before insertion).
**Anodization (Additive)**	Electrochemical oxidation using specific electrolytes (e.g., HF and H_2_SO_4_) and applied voltage to grow self-organized TiO_2_/ZrO_2_ nanotubes.	High surface-to-volume ratio; tunable nanotube diameter; allows for localized drug/ion loading within the tubular structures.	Nanotubular structures can be brittle under high shear stress during implant insertion; requires strict optimization to avoid coating delamination.
**CaP/HA Coatings (Additive)**	Deposition of Calcium Phosphate or Hydroxyapatite via plasma spraying, pulsed laser deposition, or biomimetic precipitation.	High osteoconductivity; chemically mimics the inorganic phase of native bone; promotes direct chemical bonding.	Plasma-sprayed thick coatings are highly prone to cracking and delamination under cyclic fatigue; unpredictable in vivo resorption rates.
**Biofunctionalization (Peptides/Ions)**	Dip-coating, covalent tethering, or electrochemical doping to immobilize RGD sequences, BMPs, or ions (Sr^2+^, Mg^2+^, Cu^2+^).	Active osteoinstruction; targeted molecular signaling; potential dual-action properties (e.g., simultaneous osseointegration and antimicrobial action).	Complex and costly manufacturing; severe regulatory hurdles; risk of initial “burst release” and localized cytotoxicity; long-term peptide stability in vivo remains uncertain.

**Table 3:** Hierarchical design principles for advanced endosseous implants and their corresponding biological and clinical applications.

Hierarchical Level	Design Principle	Technique	Clinical/Biological Application	Key Metrics (Range/Mean)
**Macro/Micro**	Mechanical Interlocking	Sandblasting/Acid-Etching (SLA)	Primary stability; bone-to-implant contact (BIC)	N.A.
**Nanostructural**	ECM Emulation	Anodization (TiO_2_ nanotubes)	Protein adsorption; MSC recruitment and adhesion	N.A.
**Molecular**	Biochemical Signaling	Biomolecular grafting (RGD, BMP-2, Mg^2+^/Sr^2+^)	Osteo-instructive signaling; accelerated mineralization	N.A.
**Mechanical**	Compliance Matching	Ti-Zr alloy/PEEK composites	Young’s modulus optimization; stress shielding mitigation	N.A.

**Table 4:** Clinical advantages associated with the transition to osteoinstructive biomimetic surfaces.

Clinical Parameter	Description
**Enhanced Healing Kinetics**	Increased bioactivity reduces the latency period between implant placement and prosthetic loading, optimizing the surgical workflow.
**Biological Integrity**	A robust, biologically integrated interface reduces the risk of early and late implant failure.
**Proactive Defence**	Nanostructured surfaces inhibit biofilm formation, providing a physiological defence against peri-implantitis and soft tissue recession.

**Table 5:** Summary of clinical outcomes for Ti-15Zr (Roxolid™) narrow-diameter implants reported in systematic reviews and clinical studies.

Clinical Outcome	Evidence	Key Metrics (Range/Mean)
Survival Rate (1 yr)	≥98.4% for Ti-Zr 3.3 mm narrow-diameter implants (NDIs)	N.A.
Survival Rate (2 yr)	≥97.7%; marginal bone loss (MBL) ≈ 0.41 mm	N.A.
Fracture Resistance	No implant body fractures reported in primary studies despite narrow diameter	N.A.
Soft Tissue Integration	Superior attachment vs. machined or ceramic surfaces; robust biological seal	N.A.
Immunomodulation	M2 macrophage polarization; reduced IL-6, TNF-α, IL-1β; upregulated IL-10 and TGF-β1	N.A.
Osteoblast Activity	Increased ALP activity and Osteocalcin (OC) expression vs. cpTi	N.A.

## Data Availability

No new data were created or analyzed in this study.

## Abbreviations

The following abbreviations are used in this manuscript:

ALP	Alkaline Phosphatase
BII	Bone-Implant Interface
BIC	Bone-to-Implant Contact
BMP	Bone Morphogenetic Protein
BMP-2	Bone Morphogenetic Protein-2
BMP-4	Bone Morphogenetic Protein-4
BMP-7	Bone Morphogenetic Protein-7
BoP	Bleeding on Probing
BSP	Bone Sialoprotein
CaP	Calcium Phosphate
cpTi	Commercially Pure Titanium
ECM	Extracellular Matrix
ERK	Extracellular signal-Regulated Kinase
FAK	Focal Adhesion Kinase
GRGD	Gly-Arg-Gly-Asp (peptide sequence)
HA	Hydroxyapatite
IDCT	Innovative Dip-Coating Technique
IL-1β	Interleukin-1 beta
IL-6	Interleukin-6
IL-10	Interleukin-10
MAPK	Mitogen-Activated Protein Kinase
MBL	Marginal Bone Loss
modSLA	Modified Sand-blasted Large-grit Acid-etched surface
MSC	Mesenchymal Stem Cell
MSN	Micro-/Submicro-/Nanostructured (surface)
NDI	Narrow-Diameter Implant
OC	Osteocalcin
OPG	Osteoprotegerin
PDGF	Platelet-Derived Growth Factor
PEEK	Polyether Ether Ketone
PI3K	Phosphoinositide 3-Kinase
RANKL	Receptor Activator of Nuclear factor Kappa-B Ligand
RGD	Arg-Gly-Asp (peptide sequence)
RUNX2	Runt-related transcription factor 2
Sa	Arithmetic Mean Height (surface roughness parameter)
SEM	Scanning Electron Microscopy
SLA	Sand-blasted Large-grit Acid-etched (surface)
SMAD	Suppressor of Mothers Against Decapentaplegic (signaling proteins)
Sr^2+^	Strontium ion
Mg^2+^	Magnesium ion
Sz	Maximum Peak-to-Valley Height (surface roughness parameter)
TGF-β	Transforming Growth Factor-beta
TGF-β1	Transforming Growth Factor-beta 1
Ti-Zr	Titanium–zirconium (alloy)
TiO_2_	Titanium Dioxide
TNF-α	Tumor Necrosis Factor-alpha
VEGF	Vascular Endothelial Growth Factor
XPS	X-ray Photoelectron Spectroscopy
ZrO_2_	Zirconium Dioxide
